# Influence of perceived barriers and facilitators for physical activity on physical activity levels in patients with rheumatoid arthritis or spondyloarthritis: a cross-sectional study of 150 patients

**DOI:** 10.1186/s12891-021-04792-7

**Published:** 2021-10-30

**Authors:** Thomas Davergne, Rawdha Tekaya, Jérémie Sellam, Anne Tournadre, Stéphane Mitrovic, Adeline Ruyssen-Witrand, Christophe Hudry, Sabrina Dadoun, Jérôme Avouac, Bruno Fautrel, Laure Gossec

**Affiliations:** 1 Sorbonne Université, INSERM UMR-S 1136, Institut Pierre Louis d’Épidémiologie et de Santé Publique, 47-83 Boulevard de l’Hôpital, 75013 Paris, France; 2 Rheumatology Department, University of Tunis El Manar, Charles Nicolle Hospital, Tunis, Tunisie; 3 Rheumatology Department, Sorbonne Université, INSERM URMS_938, APHP, Saint-Antoine Hospital, Paris, France; 4 Rheumatology Department, University of Clermont, Auvergne, Clermont Ferrand Hospital, Clermont-Ferrand, France; 5 Internal Medicine Department, Institut Mutualiste Montsouris, Paris, France; 6 Rheumatology Department, Toulouse University Hospital, Clinical Investigation Centre CIC1436, INSERM and Paul Sabatier University Toulouse III, Toulouse, France; 7 CeSOA, MGEN, Paris, France; 8 Clinique Geoffroy Saint Hilaire, Ramsay, Paris 5, France; 9 Rheumatology Department, Université de Paris, Cochin Hospital, Paris, France; 10 Rheumatology Department, Pitié Salpêtrière Hospital, APHP, Paris, France

**Keywords:** Barriers and facilitators, Physical activity, Axial Spondyloarthritis, Rheumatoid arthritis, Psoriatic arthritis, Patient reported outcome measures

## Abstract

**Background:**

Barriers and facilitators to physical activity in inflammatory arthritis can be assessed through the Inflammatory arthritis FAcilitators and Barriers (IFAB) questionnaire. The objective was to measure the correlation between IFAB and self-reported physical activity levels.

**Methods:**

This was an international, multicentric, cross-sectional study in 2019–20. Consecutive spondyloarthritis (axSpA), rheumatoid arthritis (RA) or psoriatic arthritis (PsA) patients completed the 10-item IFAB, which ranges from − 70 to 70 with lower scores indicating more barriers. Physical activity was measured by the IPAQ-S questionnaire, steps per day collected by smartphone, and psychological readiness to change by stages of behaviour change. Spearman correlations and multivariable linear regression were calculated.

**Results:**

Of 245 patients included, 150 were analysed: 69 (46%) axSpA, 63 (42%) RA, 18 (12%) PsA. Mean age was 48.6 years (standard deviation, SD 17.1), mean disease duration 11.7 (10.1) years and 60% were women. Barriers to physical activity were moderate: mean IFAB, 6 (SD 19.2); 39 (26%) patients scored less than − 5, corresponding to significant barriers. The mean physical activity was 2837 (SD 2668, median 1784) MET-minutes per week. The IPAQ-S questionnaire was correlated with the IFAB (rho 0.28, *p* < 0.001), as well as the stage of behaviour change (rho 0.35, *p* < 0.001) though not with steps per day. Multivariable analyses were confirmatory.

**Conclusion:**

Perceived barriers and facilitators to physical activity were correlated with physical activity, indicating that targeting patients with high barriers and low facilitators to physical activity could be an effective option to improve physical activity levels.

**Trial registration:**

ClinicalTrial NCT04426747. Registered 11 June 2020 - Retrospectively registered.

**Supplementary Information:**

The online version contains supplementary material available at 10.1186/s12891-021-04792-7.

## Significance and innovation

- Patients with inflammatory arthritis are more prone to physical inactivity than the general population but derive specific benefits from regular physical activity.
- In this study, a majority (67%) of patients with inflammatory arthritis reported a low to moderate levels of physical activity and only 27% reached the recommended amount of physical activity as defined by 7000 steps per day.
- In this population of 150 patients with inflammatory arthritis, a link was observed between a global score of barriers and facilitators and physical activity levels collected through IPAQ-S.
- This questionnaire could be a practical tool to use in clinical practice and in research to address perceived barriers and facilitators to physical activity in order to increase the physical activity levels of patients with inflammatory arthritis.

## Background

Physical inactivity has been identified as the fourth leading risk factor for global mortality around the world [1]. Moreover, the positive effects of physical activity on health, wellbeing and reduced mortality are widely established and documented for all ages [2–5].

Patients with inflammatory arthritis (IA), such as axial spondyloarthritis (axSpA), rheumatoid arthritis (RA) or psoriatic arthritis (PsA) are more prone to physical inactivity than the general population [6, 7]. Despite this, patients with IA derive specific benefits from regular physical activity [8–10]. In addition, patients with IA are at risk of other co-morbidities such as cardio-vascular diseases which can also be positively influenced by physical activity [11, 12]. However, increasing physical activity in IA patients is a challenge [13]. Lifestyle changes should be addressed by a global approach taking into account behavioural barriers to increase chances of success [14].

Barriers and facilitators to physical activity are key elements to understand physical activity behaviour in rheumatic diseases [15–18]. These elements can be classified as symptoms of the condition, social or physical environment of the person, and/or psychological status [19]. A Questionnaire for Inflammatory arthritis patients assessing FAcilitators and Barriers to physical activity (IFAB) has been recently developed and validated in patients with IA [20]. The relevance of a score to assess barriers and facilitators would be increased if a link was shown with physical activity behaviours. Furthermore, such a link would allow to consider alternative and enhanced approaches to physical activity assessment and interventions. Previous studies have sought to determine which factors influence physical activity levels in patients with IA. They showed a link between physical activity and general or arthritis-specific barrier limitations but without considering a global score including barriers and facilitators [21–23].

Therefore, the objective was to measure the correlation between barriers and facilitators, assessed through the IFAB questionnaire, and self-reported physical activity levels. We also explored other markers of physical activity, through stages of behaviour change and steps per day indicated by smartphones.

## Methods

### Study design

The ImBAIA study was an international, multicentric, cross-sectional study in a usual-care setting, performed in secondary and tertiary care hospitals in France (10 centres) and in Tunisia (one centre), between October 2019 and June 2020 (ClinicalTrial NCT04426747) [24]. This study was approved by the ethics committee (CPP Sud-Est III, France, EudraCT 2019-A01413-54, methodology MR03 for non-interventional studies). All patients received at inclusion oral and written information, and oral consent was obtained from all participants as requested by the ethics committee, as it is a minor risk, non-interventional study. This report followed the STrengthening the Reporting of OBservational studies in Epidemiology (STROBE) statement [25].

### Participants

Inclusion criteria were: age above 18 years; definite IA confirmed by the rheumatologist based on classification criteria: axSpA (referring to the Assessment of SpondyloArthritis international Society classification criteria) [26], RA (referring to the international classification criteria of RA) [27] or PsA (referring to the ClASsification of Psoriatic ARthritis (CASPAR) criteria) [28], with no restriction for comorbidities; ability to walk, having a smartphone compatible with apps that can track steps; ability to read and write in the language of the participating country. Patients not completing the Case Report Form were excluded.

Over the recruitment period, consecutive patients with definite axSpA, RA or PsA who satisfied the inclusion criteria, seen in outpatient visits by one of the investigators, were invited to participate.

### Outcomes collected

#### Barriers and facilitators to physical activity

Barriers and facilitators to physical activity were measured through the IFAB questionnaire, validated in IA patients [20]. This questionnaire contains 10 items. The items are related to psychological status (*N* = 6), social support (*N* = 2), disease (*N* = 1) and environmental factors (N = 1). The IFAB questionnaire appears to be feasible (missing data 12%, mean completion time < 5 min), reliable (interclass coefficient 0.79), with satisfactory internal consistency (Cronbach α 0.69) and adequate concurrent validity (correlated with the modified Health Assessment Questionnaire (mHAQ); rho = 0.24) [20]. The total score ranges from − 70 to 70 with a higher score indicating a higher level of facilitators and/or a lower level of barriers. Results below − 5 were identified in the initial development as potentially justifying a targeted intervention [20].

#### Physical activity behaviour

Different indicators of physical activity behaviours were used in this study.

Self-reported levels of physical activity were collected through the International Physical Activity Questionnaire Short form (IPAQ-S) and were analysed through the number of metabolic equivalent of task (MET) minutes per week (energy expended while performing various activities throughout the whole week) [29]. To calculate MET minutes per week, the MET value given (e.g., walking = 3.3,) was multiplied by the minutes the activity was carried out and again by the number of days that that activity was undertaken. For example, if someone reports walking for 50 min 3 days a week then the total MET minutes for that activity are 3.3 X 50 X 3 = 495 Met minutes per week. The levels of physical activity were categorised as low, moderate and high following the IPAQ scoring protocol [30].

A simple question developed by the authors, assessing the feeling of doing enough activity was also used (“Do you think you do enough physical activity?”) and was scored on a 0 to 10 numeric scale, to further assess the validity of the IFAB questionnaire.

Involvement in an active lifestyle was assessed through a questionnaire, the stage of behaviour change regarding active lifestyle [31]. Active lifestyle is defined in this questionnaire as 150 min of moderate activity or 75 min of intense activity per week, according to the recommendation of the World Health Organisation [1]. Stage of behaviour change ranges 1 to 5, from precontemplation ‘(I do not engage in regular physical activity and do not intend to in the next 6 months’) to maintenance (‘I engage in regular physical activity and have been doing so for more than 6 months’) [31].

The levels of physical activity were also collected through mean daily steps per month on the last four full weeks through smartphone Apps, either installed by default (such as Health on iPhone or Samsung Health on Samsung) or installed by the patient (such as Runtastic or Fitbit) [32, 33]. The results were self-reported into the patient Case Report Form. The threshold of 7000 steps per day has been used as a recommended level of physical activity for patients with chronic disabilities [34]. The following classification was used to interpret steps per day: < 5000 = sedentary, 5000–7499 = low active, 7500–9999 = somewhat active, 10,000–12,499 = active and ≥ 12,500 = highly active [35].

#### General data collected

Other variables collected were socio-demographic data, as well as information about the underlying condition (type of IA, year of diagnosis of IA), current treatment and comorbidities, collected using the Functional Comorbidity Index (0 = no comorbidity; to 18) [36]. In patients with RA: the Disease Activity Score 28 (DAS28) was collected using the last available data for sedimentation rate or CRP concentration [37]. In patients with axSpA, disease activity was measured through the Bath Ankylosing Spondylitis Disease Activity Index (BASDAI) and through the Disease Activity Index for Psoriatic Arthritis (DAPSA) for PsA patients [38]. Function was measured via the mHAQ. Physician global assessment was assessed on a numeric scale (0–10).

### Statistical analyses

*Sample size calculation*: to demonstrate a link between the IFAB questionnaire score and IPAQ-S with a relative risk of 0.5, 144 patients were needed (with α 0.5 and β 0.20).

Descriptive statistics relied on mean (standard deviation, SD) and median values. The relationship between IFAB and physical activity, primarily using the IPAQ-S, and then exploring the stage of behavioural change and smartphone steps, was tested using Spearman’s correlation. Both total score and individual items of the IFAB questionnaire were analysed.

Two multivariable linear regressions were performed, using as dependent variable first IPAQ-S (MET-minutes per week) and the stage of behaviour change, and as explanatory variables the IFAB questionnaire. Mean daily steps per month was not used as dependent variable because not linked with IFAB score in univariate analysis. Other variables were included in the models based on statistical association in univariate analysis (*p* < 0.20) and on their clinical relevance: these were: age, gender, disease duration, physician global assessment and number of comorbidities. In this model, physical function and disease activity were not included because no statistical association were observed in univariate analysis and to avoid negative interaction with “physician’s global assessment” and “number of comorbidities. For each analysis, assumptions regarding linearity, homoscedasticity, and normality were checked. The α-level of significance was set at 0.05.

The coherence of the 3 physical activity behaviours measures was assessed by the correlation or t-test between the IPAQ-S score and mean daily steps per month, stage of behaviour and binary feeling of enough activity.

Statistical analyses were performed using R version 3.5.1. There was no imputation of missing data. There were no missing data regarding the IFAB questionnaire and physical activity (both step count and IPAQ-S) because the presence of this information was mandatory to include a person. For the rest of the data, very few missing data were observed (less than 5%) and a person with missing data was removed from the corresponding analysis. However, for mean daily steps per month on the smartphone, a correction was applied for outliers (data > 20,000 steps per day were censored at 20000).

## Results

### Participants

Of 245 patients identified to participate, 150 completed the questionnaire and were analysed: 69 (46%) axSpA, 63 (42%) RA, 18 (12%) PsA. Mean age was 48.6 years (SD 17.1), mean disease duration 11.7 (SD 10.1) years; 60% were women (Table 1). In all, 69% used bDMARDs; disease activity was moderate (Table 1).

**Table 1:** Description of the 150 patients with inflammatory arthritis

Characteristic	All patients *N* = 150
Age (years), mean (SD)	48.6 (17.1)
Women, N (%)	85 (60)
Disease duration (years), mean (SD)	11.7 (10.1)
BMI (kg/m2), mean (SD)	26.0 (5.1)
Working status, paid activity, N (%)	90 (67)
bDMARDs use, N (%)	96 (69)
Comorbidities: Functional Comorbidity Index (0–18), mean (SD)	0.9 (1.0)
mHAQ (0–3), mean (SD)	0.9 (1.0)
Disease activity score, mean (SD)	
• axSpA, *N* = 69: BASDAI (0–10)	3.6 (2.1)
• In RA (DAS28, 0.96–8.47)	2.7 (1.2)
• In PsA (DAPSA, 0- > 165)	17.5 (23.4)

*BMI* Body Mass Index, *axSpA* axial SpondyloArthritis, *RA* rheumatoid arthritis, *PsA* psoriatic arthritis, *SD* standard deviation, *BASDAI* Bath Ankylosing Spondylitis Disease Activity Index, *DAS28* Disease Activity Score, *DAPSA* Disease activity in psoriatic arthritis, *mHAQ* Modified Health Assessment Questionnaire

### Barriers and facilitators

**Fig. 1:**
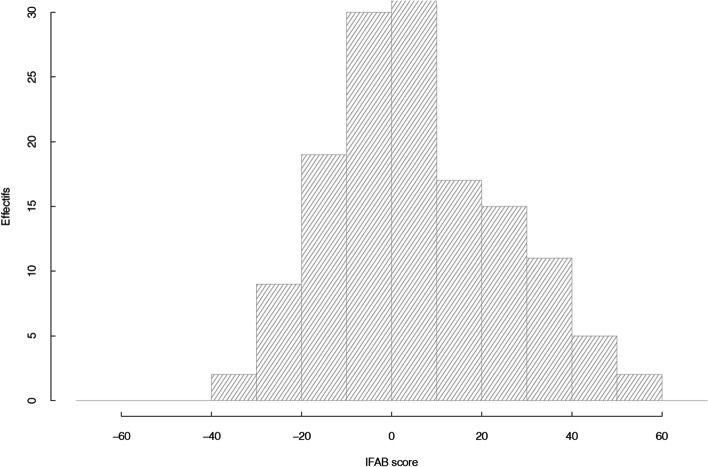
Distribution of the IFAB score measuring barriers and facilitators to physical activity in 150 patients with inflammatory arthritis

The mean score of the IFAB questionnaire was 6.0 (SD 19.2), median was 4 (Fig. 1). The two most frequent barriers or facilitators were item 1 (presence or absence of symptoms, *n* = 111, 74%) and item 9 (knowledge that physical activity is good for my health, *n* = 110, 73%). A total of 39 (26%) patients scored less than − 5 and thus could justify a targeted intervention. The IFAB score was slightly negatively associated with physical function; and furthermore, with disease activity only for patients with psoriatic arthritis (online supplementary Table 1). Multivariate analyses showed significant correlation between the IFAB score and number of comorbidities (estimate 3.6), and physician global assessment (estimate − 2.0).

### Physical activity behaviours

**Fig. 2:**
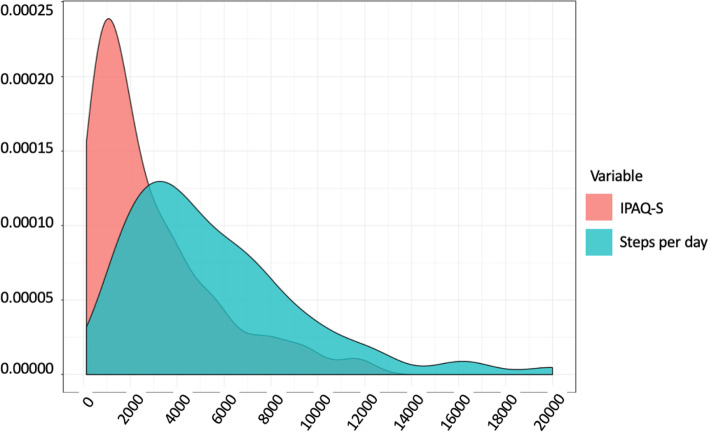
Distribution of the IPAQ-S score and mean daily steps per month in 150 patients

Self-reported physical activity through IPAQ-S was moderate (although in the high range of the moderate category; i.e., between 600 and 3000): the mean MET-minutes per week was 2837 (2668), median 1784 (Fig. 2). Levels of physical activity were high for 38% of the participants, moderate for 46% and low for 16%. In all, 56 (37%) patients reported having the feeling of doing enough activity, and 82 (54%) reported following the WHO recommendations for physical activity (stage of behaviour: action and maintenance).

Physical activity was low when collected by smartphone through mean daily steps per month: mean 5600 (SD 3797), median 4578 with 27% walking over 7000 steps per day (Fig. 2).

The IPAQ-S was slightly though statistically correlated to steps per day (rho 0.28, *p* < 0.001), and stage of behaviour (rho 0.21, *p* < 0.001) and was significantly higher in patients who felt sufficiently active (MET-minutes per week 4092 (SD 3210) vs 2104 (SD 1954), *p* < 0.001).

### Link between the IFAB questionnaire and physical activity behaviours

*Univariate associations:* The global score of the IFAB questionnaire was linked significantly with 3 of the 4 parameters of physical activity: IPAQ-S (rho 0.28, *p* < 0.001), stage of behaviour change (rho 0.35, *p* < 0.001) and the IFAB score was significantly higher in patients who felt sufficiently active: 13.9 (SD 16.2) vs 1.3 (SD 19.4), *p* < 0.001) (Table 2). However, there was no correlation with steps on smartphones (Table 2).

**Table 2:** correlation between IFAB questionnaire, each items and level of physical activity

Item	Correlation with IPAQ-S MET-minutes per week, rho	Score among patients not feeling active enough, mean (SD)	Score among patients feeling active enough, mean (SD)	*P* value feeling of enough PA	Correlation with stage of behavior change, rho	Correlation with mean daily steps per month, rho
Total IFAB score	0.28 ***	1.3 (19.4)	13.9 (16.2)	***	0.35 ***	0.08
Item 1	0.29 ***	−3.1 (4.9)	0.3 (5.2)	***	0.16 *	0.16 *
Item 2	0.06	− 0.1 (4.3)	−1.4 (3.8)	–	0.02	0.05
Item 3	0.08	1.1 (4.9)	2.0 (4.3)	–	0.16 *	0.01
Item 4	0.13	1.5 (3.7)	2.2 (3.6)	–	0.09	−0.07
Item 5	− 0.09	−1.3 (2.5)	−0.9 (2.4)	–	0.10	0.07
Item 6	0.22 **	−4.27 (3.4)	−2.0 (2.7)	***	0.25 ***	−0.05
Item 7	0.22 **	−2.5 (3.2)	−0.8 (2.4)	***	0.20 **	0.12
Item 8	0.16 *	4.7 (3.6)	5.9 (3.7)	–	0.26 ***	0.11
Item 9	0.15	4.1 (3.8)	5.6 (3.7)	*	0.33 ***	0.11
Item 10	0.09	2.3 (3.6)	2.9 (3.7)	–	0.17 *	0.02
Social support subgoup	0.14	2.7 (7.0)	4.0 (6.5)	–	0.27 ***	−0.03
Psychological and knowledge status subgroup	0.22 **	3.0 (11.9)	10.8 (10.7)	***	0.16 *	0.09

*SD* Standard Deviation, *PA* physical activity, Social support subgroup = items 3 and 4, Psychological and knowledge status subgroup = items 5 to 10

* = *P* < 0.05, ** = *p* < 0.01, *** = *p* < 0.001

*Multivariable analyses* confirmed the significant link between physical activity through IPAQ-S and the global score of the IFAB questionnaire (beta estimate 38.7, *p* < 0.001) and between stage of behaviour change and the global score of the IFAB questionnaire (beta estimate 0.02, *p* < 0.001).

## Discussion

In this population of 150 IA patients, we observed a link between a global score of barriers and facilitators and physical activity levels collected through IPAQ-S. Furthermore, a correlation was also observed in stage of behaviour regarding active lifestyle and patients reported less barriers and/or more facilitators when feeling active enough. Similar results were observed in other studies. Brittain et al. in 2011 examined in 248 women with arthritis the link between barrier categories and participation in moderate physical activity [22]. They concluded that both arthritis-specific and general barrier limitations were the strongest predictors of self-reported moderate activity. However, this study did not consider the implication of facilitators in participation to physical activity and did not use a global score of barriers and facilitators.

This link between arthritis-specific, general barriers and self-reported moderate activity (GPAQ questionnaire) was also observed in a cross-sectional study of 96 RA patients [21].

In our study, we found that the barriers that were most correlated with all parameters of self-reported physical activity were the items related to physical condition (i.e., symptoms). This correlation was also observed in a cross-sectional study of Suh 2019 including 245 RA patients, where the 18-item Barriers to Health Activities Scale (BHAS) was compared with physical activity self-reported through IPAQ [39], and in Freid et al. in 2020 including 108 IA patients [40].

In this study, a majority of IA patients reported a moderate to low level of physical activity (62%) and only 37% patients reported having the feeling of being active enough. The mean daily steps per month collected with apps was low (5600; SD 3797) with 27% walking over 7000 steps per day [34, 35]. This means that only a minority of people with IA are undertaking the recommended amount of physical activity (as defined by 7000 steps per day) and are at risk of complications. The low level of physical activity observed in this study is coherent with other studies [6, 41, 42]. This low level is a major health concern as physical activity is associated with a wide variety of benefits such as a decrease of cardiovascular risk, a decrease of disease activity and an increase of physical function [8–11].

A quarter of patients (26%) scored less than − 5 on the IFAB questionnaire (the lower 25% of the group) and could justify targeted intervention. The most common barrier or facilitator was the presence or absence of symptoms. Indeed, symptoms such as pain, fatigue and stiffness are highly prevalent in IA patients and lead to disability. Controlling symptoms might be a relevant strategy to enable regular physical activity. The second most reported determinant of physical activity was the knowledge that physical activity is good for health. This item was correlated with the stage of behaviour change and the feeling of being active enough (but not with IPAQ-S and mean daily steps per week) and could be an easy target to modify through patient education. This underlines the importance of physical activity education and its health benefits. It appears that the IFAB questionnaire could be a practical tool to use in clinical practice and in research. Addressing perceived barriers and facilitators to physical activity by using this questionnaire may be key to increasing the physical activity levels of IA patients.

Similarly in a cross-sectional study of 2002, Bell et al. studied in of 137 IA patients the link between physical activity objectively measured with thigh worn physical activity monitor and exercise beliefs questionnaire [23]. They observed that attending an exercise facility in the community and low role limitations due to physical health predicted low physical activity. Following the physical activity guideline was linked with low role limitations due to emotional problems, higher physical fitness and healthier exercise attitudes and beliefs.

This study has strengths and weaknesses. First of all, we observed a correlation between barriers and facilitators and self-reported physical activity but not with mean daily steps per month. This could be explained by the fact that a wide variety of physical activities are not covered by steps per day through smartphone, such as swimming or arm movement [43]. A substantial part of the physical activity can be related to domestic activity such as gardening or cleaning. These activities are generally not well captured by the smartphone, leading to an unrepresentative measure of physical activity [44].

Secondly, only 61% (150/245) of patients included in this study had analysable data. This exclusion rate is due to the method of recruiting participants and in particular to the difficulty of using the electronic form. Furthermore, the demographics of the study sample, such as being mainly White, middle class women, limits the generalizability of the findings. However, this study included IA patients through 3 different conditions (axSpA, RA, PsA). These 3 conditions are the most prevalent inflammatory joint and spine diseases. They share common characteristics such as pain and fatigue, swelling in the joints or axial stiffness, systemic manifestations and can potentially lead to structural changes in joint or spine with loss of function [45, 46]. Finally, one of the strengths of this study is the use of a validated questionnaire providing a global score of barriers and facilitators to physical activity.

## Conclusions

In conclusion, perceived barriers and facilitators to physical activity were correlated with physical activity, indicating that targeting IA patients with high barriers and low facilitators to physical activity could be an effective option to improve physical activity levels. Addressing perceived barriers and facilitators to physical activity by using the IFAB questionnaire may be key to increasing the physical activity levels of IA patients.

## Supplementary Information

**Additional file 1 **: **Online Supplementary Table**. Link between IFAB questionnaire and other variables.

## Data Availability

The datasets generated during and/or analysed during the current study are available from the corresponding author on reasonable request. The codes used in R software during the current study are available from the corresponding author on reasonable request.

## Abbreviations

axSpA: Axial spondyloarthritis
BAS28: Disease Activity Score 28
BASDAI: Bath Ankylosing Spondylitis Disease Activity Index
BHAS: Barriers to Health Activities Scale questionnaire
CASPAR: ClASsification of Psoriatic ARthritis criteria
DAPSA: Disease Activity Index for Psoriatic Arthritis
GPAQ: General barriers and self-reported moderate activity questionnaire
IA: Inflammatory arthritis
IFAB: FAcilitators and Barriers to physical activity questionnaire
IPAQ-S: International Physical Activity Questionnaire Short form
MET: Metabolic equivalent of task
mHAQ: Modified Health Assessment Questionnaire
PsA: Psoriatic arthritis
RA: Rheumatoid arthritis
STROBE: STrengthening the Reporting of OBservational studies in Epidemiology statement

## References

[1] WHO (2018). Global action plan on physical activity 2018–2030: more active people for a healthier world.

[2] Almeida OP, Khan KM, Hankey GJ, Yeap BB, Golledge J, Flicker L (2014). 150 minutes of vigorous physical activity per week predicts survival and successful ageing: a population-based 11-year longitudinal study of 12 201 older Australian men. Br J Sports Med.

[3] Orrow G, Kinmonth A-L, Sanderson S, Sutton S (2012). Effectiveness of physical activity promotion based in primary care: systematic review and meta-analysis of randomised controlled trials. BMJ..

[4] Evenson KR, Wen F, Herring AH (2016). Associations of accelerometry-assessed and self-reported physical activity and sedentary behavior with all-cause and cardiovascular mortality among US adults. Am J Epidemiol.

[5] Kahn EB, Ramsey LT, Brownson RC, Heath GW, Howze EH, Powell KE (2002). The effectiveness of interventions to increase physical activity. A systematic review. Am J Prev Med.

[6] O’Dwyer T, Rafferty T, O’Shea F, Gissane C, Wilson F (2014). Physical activity guidelines: is the message getting through to adults with rheumatic conditions?. Rheumatology..

[7] Sinnathurai P, Capon A, Buchbinder R, Chand V, Henderson L, Lassere M (2018). Cardiovascular risk management in rheumatoid and psoriatic arthritis: online survey results from a national cohort study. BMC Rheumatol.

[8] Peters MJL, Symmons DPM, McCarey D, Dijkmans BAC, Nicola P, Kvien TK (2010). EULAR evidence-based recommendations for cardiovascular risk management in patients with rheumatoid arthritis and other forms of inflammatory arthritis. Ann Rheum Dis.

[9] van der Heijde D, Ramiro S, Landewé R, Baraliakos X, den Bosch FV, Sepriano A (2017). 2016 update of the ASAS-EULAR management recommendations for axial spondyloarthritis. Ann Rheum Dis.

[10] Wendling D, Lukas C, Prati C, Claudepierre P, Gossec L, Goupille P (2018). 2018 update of French Society for Rheumatology (SFR) recommendations about the everyday management of patients with spondyloarthritis. Joint Bone Spine..

[11] Cook MJ, Bellou E, Bowes J, Sergeant JC, O’Neill TW, Barton A (2018). The prevalence of co-morbidities and their impact on physical activity in people with inflammatory rheumatic diseases compared with the general population: results from the UK Biobank. Rheumatology (Oxford).

[12] Liff MH, Hoff M, Fremo T, Wisløff U, Thomas R, Videm V (2019). Cardiorespiratory fitness in patients with rheumatoid arthritis is associated with the patient global assessment but not with objective measurements of disease activity. RMD Open.

[13] Niedermann K, Nast I, Ciurea A, Vlieland TV, van Bodegom-Vos L (2019). Barriers and facilitators of vigorous cardiorespiratory training in axial spondyloarthritis: surveys among patients, physiotherapists, and rheumatologists. Arthritis Care Res.

[14] Knittle K, De Gucht V, Hurkmans E, Peeters A, Ronday K, Maes S (2015). Targeting motivation and self-regulation to increase physical activity among patients with rheumatoid arthritis: a randomised controlled trial. Clin Rheumatol.

[15] Kanavaki AM, Rushton A, Efstathiou N, Alrushud A, Klocke R, Abhishek A (2017). Barriers and facilitators of physical activity in knee and hip osteoarthritis: a systematic review of qualitative evidence. BMJ Open.

[16] Belsi A, Papi E, McGregor AH (2016). Impact of wearable technology on psychosocial factors of osteoarthritis management: a qualitative study. BMJ Open.

[17] Martin Ginis KA, Ma JK, Latimer-Cheung AE, Rimmer JH (2016). A systematic review of review articles addressing factors related to physical activity participation among children and adults with physical disabilities. Health Psychol Rev.

[18] Rimmer JH, Marques AC (2012). Physical activity for people with disabilities. Lancet.

[19] Davergne T, Moe RH, Fautrel B, Gossec L (2019). Thu0716-Hpr major barriers and facilitators to physical activity in rheumatoid arthritis are related to physical and psychological health, setting and social environmental factors: a systematic literature review. Ann Rheum Dis.

[20] Davergne T, Moe RH, Fautrel B, Gossec L (2020). Development and initial validation of a questionnaire to assess facilitators and barriers to physical activity for patients with rheumatoid arthritis, axial spondyloarthritis and/or psoriatic arthritis. Rheumatol Int.

[21] Tan XL, Pugh G, Humby F, Morrissey D (2019). Factors associated with physical activity engagement among adults with rheumatoid arthritis: a cross-sectional study. Musculoskelet Care.

[22] Brittain DR, Gyurcsik NC, McElroy M, Hillard SA (2011). General and arthritis-specific barriers to moderate physical activity in women with arthritis. Womens Health Issues.

[23] Bell K, Hendry G, Steultjens M. Physical activity and sedentary behaviour in people with inflammatory joint disease: a cross sectional study. Arthritis Care Res. 2020;n/a n/a. 10.1002/acr.24438. 10.1002/acr.24438 PMC11497293 32886866

[24] Davergne T, Tekaya R, Deprouw C, Sellam J, Tournadre A, Mitrovic S (2021). To apply the recent EULAR recommendations, more knowledge on adherence patterns to medication and to physical activity is needed. Joint Bone Spine.

[25] von Elm E, Altman DG, Egger M, Pocock SJ, Gøtzsche PC, Vandenbroucke JP (2008). The Strengthening the reporting of observational studies in epidemiology (STROBE) statement: guidelines for reporting observational studies. J Clin Epidemiol.

[26] Rudwaleit M, van der Heijde D, Landewé R, Listing J, Akkoc N, Brandt J (2009). The development of assessment of SpondyloArthritis international society classification criteria for axial spondyloarthritis (part II): validation and final selection. Ann Rheum Dis.

[27] Aletaha D, Neogi T, Silman AJ, Funovits J, Felson DT, Bingham CO (2010). 2010 rheumatoid arthritis classification criteria: an American College of Rheumatology/European league against rheumatism collaborative initiative. Arthritis Rheumatism.

[28] Taylor W, Gladman D, Helliwell P, Marchesoni A, Mease P, Mielants H (2006). Classification criteria for psoriatic arthritis: development of new criteria from a large international study. Arthritis Rheumatism..

[29] Lee PH, Macfarlane DJ, Lam T, Stewart SM (2011). Validity of the international physical activity questionnaire short form (IPAQ-SF): a systematic review. Int J Behav Nutr Phys Act.

[30] IPAQ scoring protocol - International Physical Activity Questionnaire. https://sites.google.com/site/theipaq/scoring-protocol. Accessed 16 Dec 2020.

[31] Marcus BH, Selby VC, Niaura RS, Rossi JS (1992). Self-efficacy and the stages of exercise behavior change. Res Q Exerc Sport.

[32] Case MA, Burwick HA, Volpp KG, Patel MS (2015). Accuracy of smartphone applications and wearable devices for tracking physical activity data. JAMA..

[33] Duncan MJ, Wunderlich K, Zhao Y, Faulkner G (2018). Walk this way: validity evidence of iphone health application step count in laboratory and free-living conditions. J Sports Sci.

[34] Tudor-Locke C, Craig CL, Aoyagi Y, Bell RC, Croteau KA, De Bourdeaudhuij I (2011). How many steps/day are enough? For older adults and special populations. Int J Behav Nutr Phys Act.

[35] Tudor-Locke C, Craig CL, Thyfault JP, Spence JC (2013). A step-defined sedentary lifestyle index: <5000 steps/day. Appl Physiol Nutr Metab.

[36] Groll DL, Bombardier C, Wright JG, To T (2005). The development of a comorbidity index with physical function as the outcome. J Clin Epidemiol.

[37] Prevoo ML, van’t Hof MA, Kuper HH, van Leeuwen MA, van de Putte LB, van Riel PL (1995). Modified disease activity scores that include twenty-eight-joint counts. Development and validation in a prospective longitudinal study of patients with rheumatoid arthritis. Arthritis Rheum.

[38] Schoels MM, Aletaha D, Alasti F, Smolen JS (2016). Disease activity in psoriatic arthritis (PsA): defining remission and treatment success using the DAPSA score. Ann Rheum Dis.

[39] Suh C-H, Jung J-Y, Oh H, Boo S (2019). Evaluation of factors affecting the levels of physical activity in patients with rheumatoid arthritis: a cross-sectional study. Clin Rheumatol.

[40] Freid LM, Ogdie A, Baker JF (2020). Physical activity patterns in people with inflammatory arthritis indicate they have not received recommendation-based guidance from health care providers. ACR Open Rheumatol.

[41] Swinnen TW, Scheers T, Lefevre J, Dankaerts W, Westhovens R, de Vlam KL (2014). Physical activity assessment in patients with axial Spondyloarthritis compared to healthy controls: a technology-based approach. PLoS One.

[42] Jacquemin C, Servy H, Molto A, Sellam J, Foltz V, Gandjbakhch F (2018). Physical activity assessment using an activity tracker in patients with rheumatoid arthritis and axial spondyloarthritis: prospective observational study. JMIR mHealth uHealth.

[43] Bassett DR, Toth LP, LaMunion SR, Crouter SE (2017). Step counting: a review of measurement considerations and health-related applications. Sports Med.

[44] Westerterp KR (2009). Assessment of physical activity: a critical appraisal. Eur J Appl Physiol.

[45] Feld J, Chandran V, Haroon N, Inman R, Gladman D (2018). Axial disease in psoriatic arthritis and ankylosing spondylitis: a critical comparison. Nat Rev Rheumatol.

[46] Schett G, Gravallese E (2012). Bone erosion in rheumatoid arthritis: mechanisms, diagnosis and treatment. Nat Rev Rheumatol.

