# Supplementary material for: Influence of perceived barriers and facilitators for physical activity on physical activity levels in patients with rheumatoid arthritis or spondyloarthritis: a cross-sectional study of 150 patients
Source: BMC Musculoskelet Disord. 2021 Oct 30;22:915. doi: 10.1186/s12891-021-04792-7 (PMC8556961; doi:10.1186/s12891-021-04792-7)
Supplement: Supplementary file 1 — Additional file 1 : Online Supplementary Table. Link between IFAB questionnaire and other variables. [file 12891_2021_4792_MOESM1_ESM.docx]

Online supplementary Table 1: link between IFAB questionnaire and other variables

| Item | Link with total IFAB score, rho |
| --- | --- |
| Age | -0.04 |
| Ongoing biotherapy | 0.05 |
| BMI | -0.12 |
| Gender | 2.2$ |
| Disease duration | 0.03 |
| Function (mHAQ) | -0.20 ** |
| Physician global assessment | -0.15 |
| Perception of impact by physician | -0.07 |
| Number of comorbidities | 0.08 |
| Disease activity (DAS28) | -0.03 |
| Disease activity (BASDAI) | -0.19 |
| Disease activity (DAPSA) | -0.44 *** |
| Type of disease, | 1.14£ |
| BMI: Body Mass Index, mHAQ: Modified Health Assessment Questionnaire, BASDAI: Bath Ankylosing Spondylitis Disease Activity Index, DAS28: Disease Activity Score, DAPSA: Disease activity in psoriatic arthritis, *=P<0.05, **=p<0.01, ***=p<0.001, $=mean difference, £=estimage of linear regression. | |
